# Supplementary material for: From sequence to enzyme mechanism using multi-label machine learning
Source: BMC Bioinformatics. 2014 May 19;15:150. doi: 10.1186/1471-2105-15-150 (PMC4229970; doi:10.1186/1471-2105-15-150)
Supplement: Additional file 2 — Java code of ml2db. Additional file ml2db_code.tar.gz contains the Java source code to run the multi-label machine learning experiments and save the results to database. The code’s Javadoc is included. [file 1471-2105-15-150-S2.zip › additional file 2/ml2db/ml2db/doc/mulan/evaluation/class-use/Evaluation.html]

Uses of Class mulan.evaluation.Evaluation


JavaScript is disabled on your browser.


- Overview
- Package
- Class
- Use
- Tree
- Deprecated
- Index
- Help

- Prev
- Next

- Frames
- No Frames

- All Classes

## Uses of Class mulan.evaluation.Evaluation

- Packages that use Evaluation

  | Package | Description |
  | --- | --- |
  |  |  |
  | --- | --- |
  | mulan.evaluation |  |
- - ### Uses of Evaluation in mulan.evaluation

    Methods in mulan.evaluation that return Evaluation

    | Modifier and Type | Method and Description |
    | --- | --- |
    |  |  |
    | --- | --- |
    | `Evaluation` | Evaluator.`evaluate(MultiLabelLearner learner, MultiLabelInstances data)` Evaluates a `MultiLabelLearner` on given test data set. |
    | `Evaluation` | Evaluator.`evaluate(MultiLabelLearner learner, MultiLabelInstances data, java.util.List<Measure> measures)` Evaluates a `MultiLabelLearner` on given test data set using specified evaluation measures |

    Methods in mulan.evaluation with parameters of type Evaluation

    | Modifier and Type | Method and Description |
    | --- | --- |
    |  |  |
    | --- | --- |
    | `void` | MultipleEvaluation.`addEvaluation(Evaluation evaluation)` Adds an evaluation results to the list of evaluations |

    Constructors in mulan.evaluation with parameters of type Evaluation

    Constructor and Description

    `MultipleEvaluation(Evaluation[] someEvaluations,
    MultiLabelInstances data)`

    Constructs a new object with given array of evaluations

- Overview
- Package
- Class
- Use
- Tree
- Deprecated
- Index
- Help

- Prev
- Next

- Frames
- No Frames

- All Classes
